# Natural Heart Regeneration in a Neonatal Rat Myocardial Infarction Model

**DOI:** 10.3390/cells9010229

**Published:** 2020-01-16

**Authors:** Hanjay Wang, Michael J. Paulsen, Camille E. Hironaka, Hye Sook Shin, Justin M. Farry, Akshara D. Thakore, Jinsuh Jung, Haley J. Lucian, Anahita Eskandari, Shreya Anilkumar, Matthew A. Wu, Mariana C. Cabatu, Amanda N. Steele, Lyndsay M. Stapleton, Yuanjia Zhu, Y. Joseph Woo

**Affiliations:** 1 Department of Cardiothoracic Surgery, Stanford University, Stanford, CA 94305, USA; hanjay@stanford.edu (H.W.); mpaulsen@stanford.edu (M.J.P.); c.e.hironaka@gmail.com (C.E.H.); hyesook@stanford.edu (H.S.S.); justinfarry@stanford.edu (J.M.F.); athakore@stanford.edu (A.D.T.); jinsuhj@stanford.edu (J.J.); hlucian@stanford.edu (H.J.L.); aeskandari@ucla.edu (A.E.); shreya97@stanford.edu (S.A.); mw26511@gmail.com (M.A.W.); mariana.c.cabatu@vanderbilt.edu (M.C.C.); ansteele@stanford.edu (A.N.S.); lyndsays@stanford.edu (L.M.S.); yuanjiaz@stanford.edu (Y.Z.); 2 Stanford Cardiovascular Institute, Stanford University, Stanford, CA 94305, USA; 3 Department of Bioengineering, Stanford University, Stanford, CA 94305, USA

**Keywords:** regeneration, heart, neonate, myocardial infarction

## Abstract

Newborn mice and piglets exhibit natural heart regeneration after myocardial infarction (MI). Discovering other mammals with this ability would provide evidence that neonatal cardiac regeneration after MI may be a conserved phenotype, which if activated in adults could open new options for treating ischemic cardiomyopathy in humans. Here, we hypothesized that newborn rats undergo natural heart regeneration after MI. Using a neonatal rat MI model, we performed left anterior descending coronary artery ligation or sham surgery in one-day-old rats under hypothermic circulatory arrest (n = 74). Operative survival was 97.3%. At 1 day post-surgery, rats in the MI group exhibited significantly reduced ejection fraction (EF) compared to shams (87.1% vs. 53.0%, *p* < 0.0001). At 3 weeks post-surgery, rats in the sham and MI groups demonstrated no difference in EF (71.1% vs. 69.2%, respectively, *p* = 0.2511), left ventricular wall thickness (*p* = 0.9458), or chamber diameter (*p* = 0.7801). Masson’s trichome and picrosirius red staining revealed minimal collagen scar after MI. Increased numbers of cardiomyocytes positive for 5-ethynyl-2′-deoxyuridine (*p* = 0.0072), Ki-67 (*p* = 0.0340), and aurora B kinase (*p* = 0.0430) were observed within the peri-infarct region after MI, indicating ischemia-induced cardiomyocyte proliferation. Overall, we present a neonatal rat MI model and demonstrate that newborn rats are capable of endogenous neocardiomyogenesis after MI.

## 1. Introduction

Ischemic heart disease represents one of the greatest threats to human health, affecting over 150 million people worldwide and accounting for 10 million deaths globally per year [1]. In the United States, the cost of treating ischemic heart disease may increase by 100% over the next two decades [2]. New pharmacologic therapies and coronary revascularization strategies are being developed [3,4], but many patients nevertheless progress to heart failure and succumb to ischemic cardiomyopathy despite optimal treatment of MI [5]. Thus, there exists a significant unmet clinical need for the development of novel therapeutic strategies to prevent and treat ischemic heart failure after MI.

Heart regeneration has been the focus of extensive recent research [6]. After MI, insufficient tissue perfusion results in cardiomyocyte cell death, followed by replacement of non-viable cardiac muscle with collagen scar, leading to a thinned left ventricle (LV) wall [7]. The LV gradually undergoes compensatory remodeling characterized by ventricular dilatation, but ultimately fails, resulting in poor contractility and reduced cardiac output. Exogenous delivery of stem cells [8,9], engineered tissues [10,11,12,13,14], cytokines [15,16,17], and growth factors [18,19] to injured myocardium have demonstrated promising results for activating intrinsic myocardial repair pathways in various adult animal models and several human clinical trials. In response to these exogenous regenerative therapies, significant improvements in scar size and cardiac function have been observed, although complete normalization to baseline cardiac health is rare in adult animals treated at the time of MI.

Endogenous or natural heart regeneration is well-described in adult urodele amphibians (e.g., newts) and teleost fish (e.g., zebrafish), resulting impressively in minimal or no scar formation after LV apical resection injury [20,21]. Natural heart regeneration after LV apical resection has also been reported in neonatal mice and rats as well [22,23]. Using a more clinically-relevant MI injury model, however, our team and others have demonstrated that, after acute MI in newborn mice, an intrinsic neocardiomyogenic process is naturally activated in response to tissue ischemia, resulting in minimal scar formation and preservation of normal LV geometry and function at 3 weeks post-MI [24,25,26]. Recently, similar observations have been extended to newborn piglets [27,28] and possibly humans as well [29], suggesting that natural regeneration could perhaps be a neonatal phenotype that is conserved across class Mammalia.

The activation of endogenous neonatal heart regeneration pathways in adult patients after MI carries immense therapeutic potential. To date, however, natural heart regeneration after MI has only been demonstrated in neonatal mice and piglets, and it remains unknown whether other mammalian species might also exhibit this remarkable ability. Furthermore, our understanding of the mechanisms underlying neonatal heart regeneration after MI remains limited in part by the paucity of mammalian neonatal MI models by which endogenous regeneration may be studied. The discovery of new mammalian species capable of natural neonatal heart regeneration after MI would therefore be of tremendous value for the field of cardiovascular regeneration research.

Here, we built upon our prior experience with a neonatal mouse MI model [26] to develop a neonatal rat MI model. We hypothesized that, like their murine relatives, newborn rats also have the ability to undergo natural heart regeneration after MI, thereby preventing adverse LV remodeling and scarring, and preserving normal cardiac function despite significant acute ischemic injury.

## 2. Materials and Methods

### 2.1. Animal Care and Biosafety

Pregnant female Wistar rats and healthy adult male Wistar rats were obtained from Charles River Laboratories (Wilmington, MA, USA). Pregnant mothers were monitored for delivery of pups at minimum every 12 h. Neonatal pups were cared for by their nursing mother until appropriate for weaning at age 21 days old. Food and water were otherwise provided ad libitum. All experiments involving animals were performed following the United States National Institutes of Health “Guide for the Care and Use of Laboratory Animals” (8th Edition, 2011). All procedures involving animals were performed following a protocol approved by the Institutional Animal Care and Use Committee at Stanford University (Protocol 28921).

### 2.2. Neonatal Rat Myocardial Infarction Model

**Figure 1:**
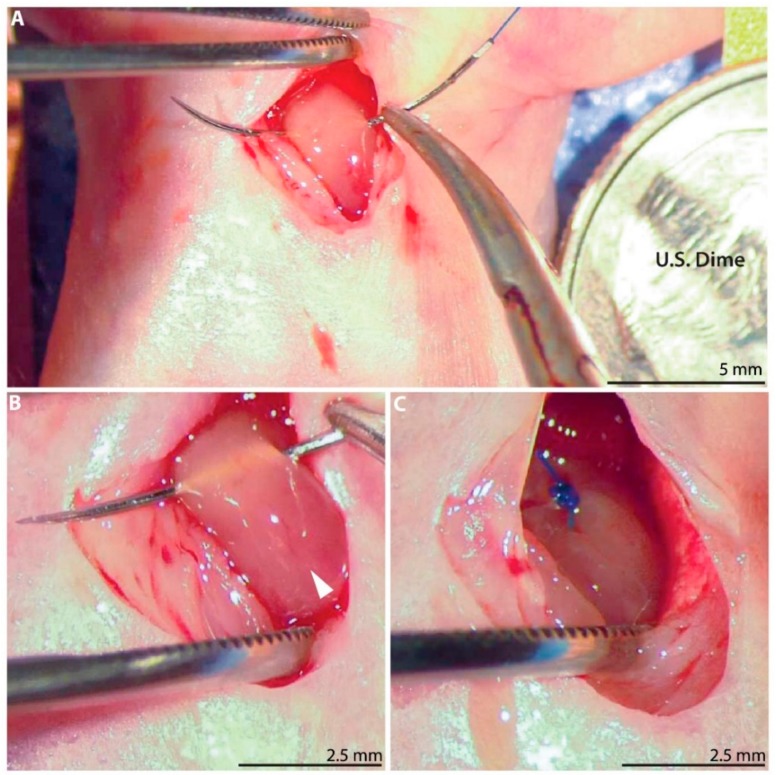
Neonatal rat myocardial infarction model. (**A**) A left anterior thoracotomy is performed to expose the heart. United States dime for scale. (**B**) A 6–0 polypropylene suture is passed below the left anterior descending (LAD) coronary artery (arrow). (**C**) The LAD artery is permanently ligated.

On postnatal day 1 (P1), defined as 12–24 h after observation of parturition, neonatal rats (*n* = 74) underwent permanent ligation of the left anterior descending (LAD) coronary artery (Figure 1A; Video S1). Both male and female neonates were included. The P1 pups were separated from their nursing mother immediately prior to surgery. Hypothermic circulatory arrest was induced via topical cooling by placing a gauze-wrapped pup in ice for 7 min [30,31]. The anesthetized pup was positioned supine on the operating table and prepped with betadine solution and ethanol. A left anterior thoracotomy was performed via the fourth intercostal space under dissecting microscope guidance to expose the heart. The LAD artery was ligated 1 mm below the left atrial appendage using a 6–0 polypropylene suture (Figure 1B,C). For sham controls, hypothermic circulatory arrest was induced, and the needle was passed through the myocardium below the LAD artery, but the suture was not tied. The chest was closed in layers using interrupted 6–0 polypropylene sutures. Just prior to skin closure, one drop of 2% lidocaine was placed in the subcutaneous tissue within the incision. The pup was then allowed to recover on a 37 °C warm plate. Once all the pups in the litter had completed surgery and were awake and active, they were cleaned and returned to maternal care.

### 2.3. Adult Rat Myocardial Infarction Model

Adult male rats (*n* = 6, 8–10 weeks old, 270–330 g) underwent permanent ligation of the LAD coronary artery as previously described [17,19,32]. The animals were anesthetized using 2–3% inhaled isoflurane (Fluriso, VetOne, Boise, ID, USA) delivered at 1 L/min, and then endotracheally intubated with a 16 G angiocatheter. Anesthesia during mechanical ventilation was maintained using continuous 1–2% isoflurane (Harvard Apparatus VentElite, Holliston, MA, USA). A left anterolateral thoracotomy was performed via the fourth intercostal space, and the pericardium was opened to expose the heart. The LAD artery was directly visualized and ligated 2 mm below the left atrial appendage using a 6–0 polypropylene suture. Consistently-sized infarcts were confirmed by snaring the suture and visually assessing the area of resulting LV pallor prior to permanently securing the suture knot. For sham controls, the needle was passed through the myocardium below the LAD artery, but the suture was not tied. The chest was closed in layers using interrupted 4–0 polypropylene sutures. After completion of surgery, the rats were extubated and recovered. Postoperative analgesia was achieved using buprenorphine (0.5 mg/kg) and carprofen (5 mg/kg).

### 2.4. Injection of 5-ethynyl-2′-deoxyuridine

A subset of neonatal rats received 5-ethynyl-2′-deoxyuridine (EdU, Thermo Fisher Scientific, Cat: A10044, Waltham, MA, USA) on postoperative days 1, 7, and 14. EdU was reconstituted to a final concentration of 2.5 mg/mL in 10% dimethyl sulfoxide in PBS and stored at −20 °C until use. Rats were anesthetized with 2–3% inhaled isoflurane and EdU (20 mg/kg) was injected subcutaneously underneath the dorsal skin. Following the procedure, the rats were recovered on a 37 °C warm plate for 20–30 min. Once all the pups in the litter were awake and active, they were cleaned and returned to maternal care.

### 2.5. Echocardiography

Transthoracic echocardiography was performed at 1 day, 1 week, 2 weeks, or 3 weeks after surgery for neonatal rats, and at 3 weeks after surgery for adult rats. Anesthesia was induced by placing the animal in a sealed chamber with 3% inhaled isoflurane. The animal was then transferred to the imaging station and positioned supine on a heated dock (37°). Anesthesia was maintained using 1–2% isoflurane delivered by nosecone. Pre-warmed gel was placed over the anterior chest. Left parasternal LV short- and long-axis images were acquired in B- and M-mode using a Vevo 2100 imaging system with ultra-high frequency linear array transducer (MicroScan MS250 13-24 MHz transducer, VisualSonics Inc., Toronto, Canada). Measurements of LV geometry, including LV wall thickness over the anterolateral LAD territory and LV internal diameter, were collected at the level of the mid-lower papillary muscles. Functional parameters, including LV ejection fraction, stroke volume, and cardiac output, were calculated using Vevo Lab software (VisualSonics Inc.).

### 2.6. Heart Explant and Sample Preparation for Histology

At 1 day, 1 week, or 3 weeks after surgery, the rats were deeply anesthetized using 4% inhaled isoflurane and euthanized by decapitation or cervical dislocation. A median sternotomy was performed and potassium chloride (1 mEq/kg) was injected into the right ventricle to induce cardiac arrest. The heart was explanted, flushed with PBS, and filled with optimum cutting temperature compound (OCT, Fisher HealthCare, Cat: 23730571, Houston, TX, USA) in a retrograde fashion. The samples were frozen in OCT using 2-methyl butane on dry ice and stored at −80 °C until use.

### 2.7. Hematoxylin and Eosin Staining

Hearts were sectioned along the LV short-axis plane at 10 μm thickness and then stained with hematoxylin and eosin (H&E) using the reagents and instructions provided in the Thermo Scientific Shandon Rapid-Chrome H&E Frozen Section Staining Kit (Thermo Fisher Scientific, Cat: 9990001). The hearts were visualized at 4× and 10× magnification using an EVOS XL Core Imaging System (Thermo Fisher Scientific, Cat: AMEX-1000).

### 2.8. Masson’s Trichrome and Picrosirius Red Staining

For neonatal hearts, three LV short-axis sections (10 μm thickness) were chosen for each heart from below the level of the ligation suture, encompassing the mid-ventricular and pre-apical levels of the LV. For adult hearts, one LV short-axis section at the level of the mid-lower papillary muscles was selected per heart.

For staining with Masson’s trichrome (American MasterTech, Cat: KTMTR2PT, Lodi, CA, USA), the tissue was thawed at 4 °C for 10 min, then rehydrated with ethanol and fixed in Bouin’s solution (Polysciences Inc., Cat: 16045-1, Warrington, PA, USA) for 1 h and 45 min at 60 °C, followed by washes with running tap water for 15 min until the tissue cleared. The tissue sections were then incubated in hematoxylin solution for 5 min, followed by Scarlet-Acid Fucshin for 15 min. The tissue sections were rinsed in deionized water, and then incubated in phosphotungstic-phosphomolybdic acid solution for 10 min. Next, the sections were stained in Aniline Blue for 5 min at room temperature, and then washed with running tap water for 2 min. The tissue sections were then incubated in 1% acetic acid for 5 min, and then washed with tap water for 2 min. Finally, the sections were dehydrated and mounted in Cytoseal (Thermo Fisher Scientific, Cat: 8310-16).

For staining with picrosirius red (Picro-Sirius Red Stain Kit-Cardiac Muscle, Abcam, Cat: ab245887, Cambridge, MA, USA), the tissue was thawed at 4 °C for 10 min, then soaked in Xylene twice for a total 15 min at room temperature. Next, the tissue was treated with sequentially diluted ethanol (100%, 96%, 80%, 70%) for 1 min each, then rinsed in deionized water and incubated in phosphomolybdic acid solution for 5 min. The tissue was then stained with picrosirius red solution for 1 h at 30 °C. Afterward, the sample was washed in 0.5% acetic acid twice for a total 3 min without agitation, then treated with sequentially concentrated ethanol (70%, 80%, 96%, 100%) for 10 s each. Finally, the tissue was soaked in Xylene twice for a total 20 min at room temperature before setting out to dry. The finished slides were imaged using an EVOS XL Core Imaging System.

### 2.9. Immunohistochemistry for Troponin, Ki-67, Aurora B Kinase

One LV short-axis section (10 μm thickness) at the level of the mid-lower papillary muscles was chosen for each heart. First, the slides were washed with PBS and fixed with 4% PFA for 10 min. The samples were then permeabilized with 0.5% PBS-Tween for 15 min, followed by blocking for 1 h with 10% fetal bovine serum. The sections were then stained with anti-cardiac troponin I primary antibody (1:200, Abcam, Cat: ab56357), along with a primary antibody for Ki-67 (1:100, Abcam, Cat: ab15580) or aurora B kinase (AurB, 1:100, Abcam, Cat: ab2254) for 90 min at 37 °C. The slides were then washed in PBS, and appropriate secondary antibodies (1:200, Abcam, Cat: ab150064 or ab150129) were applied for 45 min. Finally, the sections were incubated with DAPI (NucBlue Fixed Cell ReadyProbes Reagent, ThermoFisher Scientific, Cat: R37606) for 5 min and washed again. Microscope coverslips (Fisherbrand, Cat: 12545F, Pittsburgh, PA, USA) were added and the slides were kept at 4 °C until ready for imaging.

### 2.10. EdU Processing

One LV short-axis section (10 μm thickness) at the level of the mid-lower papillary muscles was chosen for each heart. First, 1 mL of 4% PFA was added and the sections were incubated for 15 min at room temperature. The sections were rinsed twice with 1 mL of 3% bovine serum albumin (BSA) in PBS, and then incubated in 1 mL of 0.5% PBS-Tween for 20 min. After two additional washes with 3% BSA, 0.5 mL of the Click-iT reaction cocktail was added as described in the kit protocol (Click-iT EdU Cell Proliferation Kit for Imaging, Thermo Fisher Scientific, Cat: C10339). In the dark, the sections were incubated with the Click-iT reaction cocktail for 30 min, then washed once with 3% BSA and finally twice with PBS to complete the EdU staining procedure. In preparation for troponin immunohistochemistry, the sections were incubated with anti-cardiac troponin I primary antibody (1:200, Abcam, Cat: ab47003) at 37 °C for 90 min, washed 3 times with PBS, and then incubated with an appropriate secondary antibody (1:200, Abcam, Cat: 150077) at 37 °C for 90 min. Finally, the sections were incubated with DAPI for 5 min and washed again. Microscope coverslips were added and the slides were kept at 4 °C until ready for imaging.

### 2.11. Image Acquisition and Cell Quantification

Using an inverted Zeiss LSM 780 multiphoton laser scanning confocal microscope (Zeiss, Oberkochen, Germany), heart sections were visualized using 10× and 20× objectives. Images were captured and processed using Zeiss Zen software. Image analysis was performed manually using the Z-stack images in the Zen software. Cells were inspected for positive co-labeling by DAPI, troponin, and one cell proliferation marker (EdU, Ki-67, or AurB). For EdU and Ki-67, triple-positive cells were quantified in at least 3 randomly selected regions of interest (ROI, 179.4 μm × 171.3 μm) of each 20× field of view (425.1 μm × 425.1 μm). The cell counts from each ROI were then averaged to yield the final number of triple-positive cells per ROI. For AurB, triple-positive cells were quantified for the entire 20× field of view and then adjusted to represent the ROI area described above.

### 2.12. Statistical Analysis

After the initial LAD ligation surgery for both neonate and adult rats, all research team members remained blinded to the experimental group assignments until data collection was completed. Statistical analyses were performed using Stata version 14.2 (StataCorp LLC., College Station, TX, USA), and continuous variables were reported as mean ± standard deviation unless otherwise noted and compared using 2-sample *t*-tests. Because echocardiographic data was acquired at multiple timepoints for a subset of animals, a linear mixed-model analysis was performed using R 3.6.0 (with Jamovi 0.9.6.9), fit by the restricted maximum-likelihood model with unadjusted post-hoc testing for multiple comparisons [33]. The use of this model permits a repeated-measures design and also accounts for clustering effects within each individual animal. Heart rate, which is a confounding factor for many echocardiographic metrics of cardiac function, was treated as a covariate in the model, allowing analyses adjusted for heart rate. Experimental group and time point were fixed effects in the model, whereas each individual animal was the random effect. For all comparisons, a *p*-value < 0.05 was considered statistically significant. Experimental data will be made available upon reasonable request.

## 3. Results

### 3.1. LAD Ligation Generates Acute Myocardial Infarcts in Neonatal Rats

A total of 74 neonatal rats underwent sham (*n* = 30) or LAD ligation surgery (*n* = 44) at age P1. Immediate postoperative survival after all P1 surgery was 97.3% (72/74), including 100% survival (30/30) among rats in the sham group and 95.5% survival (42/44) among rats in the MI group. LAD ligation surgery duration from skin open to skin closed was typically 5 to 6 min.

Echocardiography was performed at 1 day after P1 surgery for 37 of the 74 neonatal rats in this study. Two rats in the MI group did not exhibit evidence of anterolateral LV wall motion abnormalities and were excluded. As shown in Table 1, the remaining rats in the sham (*n* = 15) and MI groups (*n* = 20) exhibited similar LV wall thickness in diastole (LVWTd, 0.59 mm vs. 0.58 mm, *p* = 0.5343) and LV diameter in diastole (LVIDd, 2.44 mm vs. 2.51 mm, *p* = 0.4551), indicating preserved cardiac geometry at 1 day after P1 MI. However, while the sham rats demonstrated hyperdynamic LV contractility with mean ejection fraction (EF) 87.1%, the rats in the MI group exhibited pronounced LV hypokinesis with mean EF 53.0% (*p* < 0.0001). LV diameter during systolic contraction (LVIDs) was also significantly greater in the P1 MI group than in the P1 sham group (1.10 mm vs. 1.86 mm, *p* < 0.0001). As a functional consequence, the rats in the MI group generated a significantly lower stroke volume (18.5 μL vs. 11.8 μL, *p* < 0.0001) and cardiac output (5.3 mL/min vs. 2.8 mL/min, *p* < 0.0001) at 1 day after P1 surgery compared to sham rats. Interestingly, the rats in the MI group exhibited a slower heart rate than the rats in the sham group at 1 day after P1 surgery (290.2 bpm vs. 238.2 bpm, *p* = 0.0042). Importantly, this variation in heart rate was not directly correlated with cardiac function in either group (Figure S1), suggesting against anesthesia-induced cardiac depression as the cause of low EF in the MI group.

**Figure 2:**
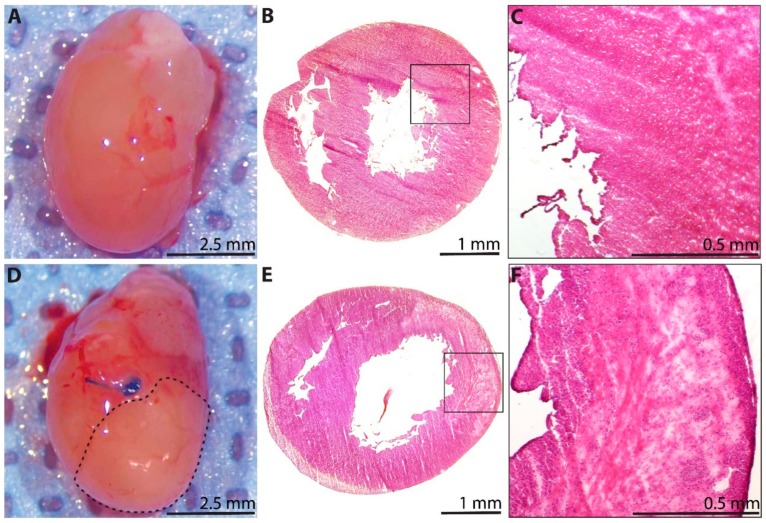
Histological assessment of the neonatal rat heart at 1 day after sham surgery or induction of myocardial infarction (MI) on postnatal day 1. (**A**) Explanted heart from the sham group. (**B**) Hematoxylin and eosin (H&E)-stained section from the sham group (*n* = 3), with (**C**) inset showing healthy myocardium, as well as a limited area of minor tissue injury from needle trauma during sham surgery (upper right corner). (**D**) Explanted heart from the MI group, showing a clear region of non-perfused myocardium below the coronary artery ligation suture (dotted line). (**E**) H&E-stained section from the MI group (*n* = 3), with (**F**) inset showing widespread areas of acute inflammatory infiltrate, severe ischemic necrosis, and tissue edema within the affected myocardium.

A subset of hearts was explanted for analysis at 1 day after P1 surgery. The explanted sham hearts appeared grossly normal (Figure 2A), with preserved tissue architecture when examined microscopically using H&E staining (*n* = 3, Figure 2B). Notably, a limited area of minor tissue damage from needle trauma during the sham procedure could be appreciated (Figure 2C). The explanted MI hearts, however, demonstrated a clear demarcation between the perfused and non-perfused myocardium below the level of the ligation suture (dotted line, Figure 2D). Furthermore, histological analyses of the post-MI hearts using H&E staining revealed widespread areas of acute inflammatory infiltrate with evidence of severe ischemic necrosis and tissue edema within the myocardium (*n* = 3, Figure 2E,F).

### 3.2. Cardiac Troponin Is Recovered by 3 Weeks after Myocardial Infarction

**Figure 3:**
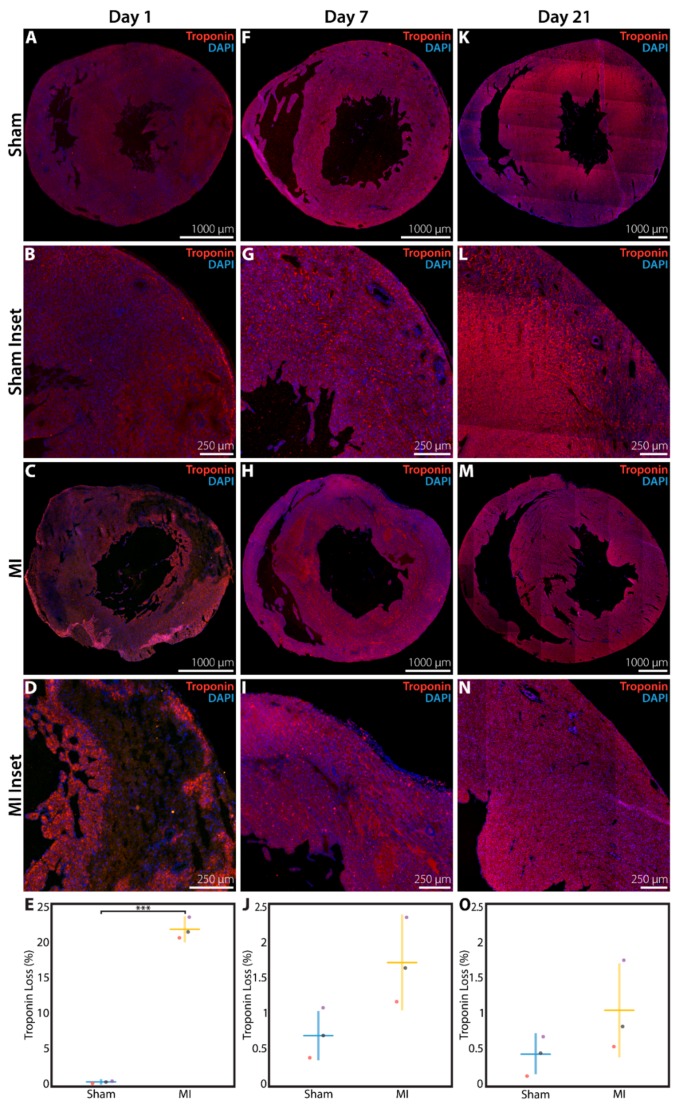
Troponin immunohistochemistry assessment of the neonatal rat heart at 1 day, 1 week, or 3 weeks after sham surgery or induction of myocardial infarction (MI) on postnatal day 1 (P1). (**A**) Sham heart at 1 day after P1 surgery (*n* = 3), with (**B**) inset. (**C**) MI heart at 1 day after P1 surgery (*n* = 3), with (**D**) inset showing significant loss of troponin^+^ cardiomyocytes. (**E**) Quantified percentage of left ventricular area devoid of troponin signal at 1 day after P1 surgery. (**F**) Sham heart at 1 week after P1 surgery (*n* = 3), with (**G**) inset. (**H**) MI heart at 1 week after P1 surgery (*n* = 3), with (**I**) inset showing minimal loss of troponin^+^ cardiomyocytes. (**J**) Quantified percentage of left ventricular area devoid of troponin signal at 1 week after P1 surgery. (**K**) Sham heart at 3 weeks after P1 surgery (*n* = 3), with (**L**) inset. (**M**) MI heart at 3 weeks after P1 surgery (*n* = 3), with (**N**) inset showing minimal loss of troponin^+^ cardiomyocytes. (**O**) Quantified percentage of left ventricular area devoid of troponin signal at 3 weeks after P1 surgery. *** indicates *p* < 0.001. Horizontal lines indicate mean. Vertical lines indicate standard deviation.

Troponin immunohistochemistry was performed to verify that LV myocardium is lost acutely after P1 MI. At 1 day after P1 surgery, the LV of rats in the sham group was homogeneously layered with troponin^+^ cardiomyocytes, with only 0.49 ± 0.09% of the total LV area devoid of troponin signal (*n* = 3, Figure 3A,B). In contrast, troponin was absent in 21.7 ± 1.5% of the total LV area (*p* < 0.0001) for rats in the MI group at 1 day after LAD ligation (*n* = 3, Figure 3C,D), with the troponin-negative region corresponding closely to the injured sector of the anterolateral wall as previously identified by H&E staining in Figure 2E,F.

At 1 week after P1 MI, a residual troponin-negative region within the anterolateral LV myocardium was still identifiable (*n* = 3, Figure 3H,I), but this area of troponin loss represented only 1.76 ± 0.63% of the total LV area. In comparison, hearts from the sham group at 1 week after P1 surgery continued to show a very small fraction of total LV area without troponin signal (0.69 ± 0.33%, *n* = 3, *p* = 0.0603, Figure 3F,G). At 3 weeks after P1 surgery, the proportion of LV area devoid of troponin signal was further decreased to 1.04 ± 0.62% for hearts in the MI group (*n* = 3, Figure 3M–N), compared to 0.44 ± 0.25% for hearts in the sham group (*n* = 3, *p* = 0.1930, Figure 3K,L). The quantified area of troponin loss within the LV at 1 day, 1 week, and 3 weeks after surgery is presented in Figure 3E, 3J, and 3O, respectively.

### 3.3. Minimal Fibrotic Scar Is Observed at 3 Weeks after Myocardial Infarction

**Figure 4:**
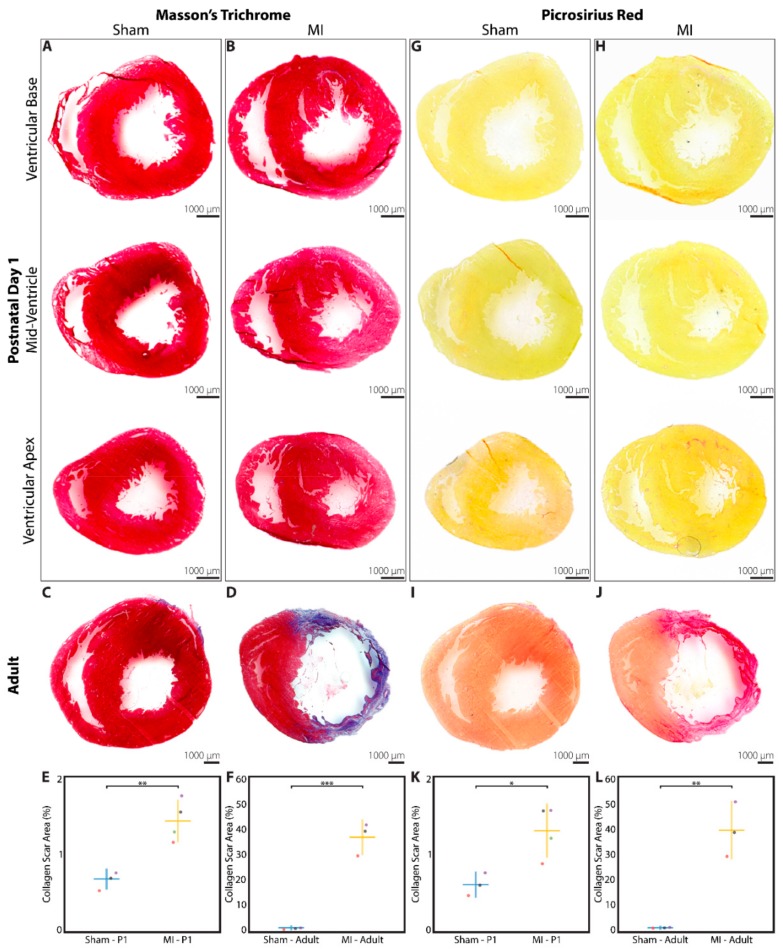
Collagen scar assessment of rat hearts at 3 weeks after sham surgery or induction of myocardial infarction (MI), performed in neonates on postnatal day 1 (P1) or in adults. Masson’s trichrome staining revealed minimal collagen scar after (**A**) P1 sham (*n* = 3) and (**B**) P1 MI surgery (*n* = 4), assessed at 3 levels below the level of suture placement. Significant collagen scar was detected after (**D**) adult MI (*n* = 3) compared to (**C**) adult sham surgery (*n* = 3). Collagen scar area relative to left ventricular area was quantified for neonates (**E**) and adults (**F**). Picrosirius red staining revealed minimal collagen scar after (**G**) P1 sham (*n* = 3) and (**H**) P1 MI surgery (*n* = 4), assessed at 3 levels below the level of suture placement. Significant collagen scar was detected after (**J**) adult MI (*n* = 3) compared to (**I**) adult sham surgery (*n* = 3). Collagen scar area relative to left ventricular area was quantified for neonates (**K**) and adults (**L**). * indicates *p* < 0.05. ** indicates *p* < 0.01. *** indicates *p* < 0.001. Horizontal lines indicate mean. Vertical lines indicate standard deviation.

At 3 weeks after P1 surgery, a subset of sham and MI hearts were explanted for analysis of collagen scar at 3 levels within the LV. Hearts from adult rats that underwent sham surgery or LAD ligation were assessed at a single level as a control comparison. Using Masson’s trichome stain and averaging across all 3 levels examined, 0.68 ± 0.11% of the total LV area stained positive for collagen in the P1 sham group (*n* = 3, Figure 4A), compared to 1.44 ± 0.25% in the P1 MI group (*n* = 4, *p* = 0.0052, Figure 4B). These data correlate well with the 3-week postoperative data from troponin immunohistochemistry previously shown in Figure 3O. In adult rats, a similar 0.65 ± 0.30% of total LV area stained positive for collagen in the sham group (*n* = 3, Figure 4C), but 37.0 ± 6.3% of LV area was positive for collagen scar in the adult MI group (*n* = 3, *p* = 0.0005, Figure 4D). The quantified scar size for neonatal and adult hearts using Masson’s trichrome stain is illustrated in Figure 4E,F, respectively.

The same sections of the neonatal and adult rat hearts were also stained with picrosirius red as a second assessment of fibrotic scar formation. Averaging across all 3 levels examined, 0.61 ± 0.15% of the LV area stained positive for collagen in the P1 sham group (*n* = 3, Figure 4G), while 1.31 ± 0.33% of the LV area stained positive for collagen in the P1 MI group (*n* = 4, *p* = 0.0193, Figure 4H). For adult rats, 0.37 ± 0.17% and 39.1 ± 10.5% of LV area stained positive for collagen in the sham and MI groups, respectively (*n* = 3, each, *p* = 0.0031, Figure 4I,J). The quantified scar size for neonatal and adult hearts using picrosirius red stain is illustrated in Figure 4K,L, respectively.

### 3.4. Increased Cardiomyocyte Proliferation Is Observed after Myocardial Infarction

**Figure 5:**
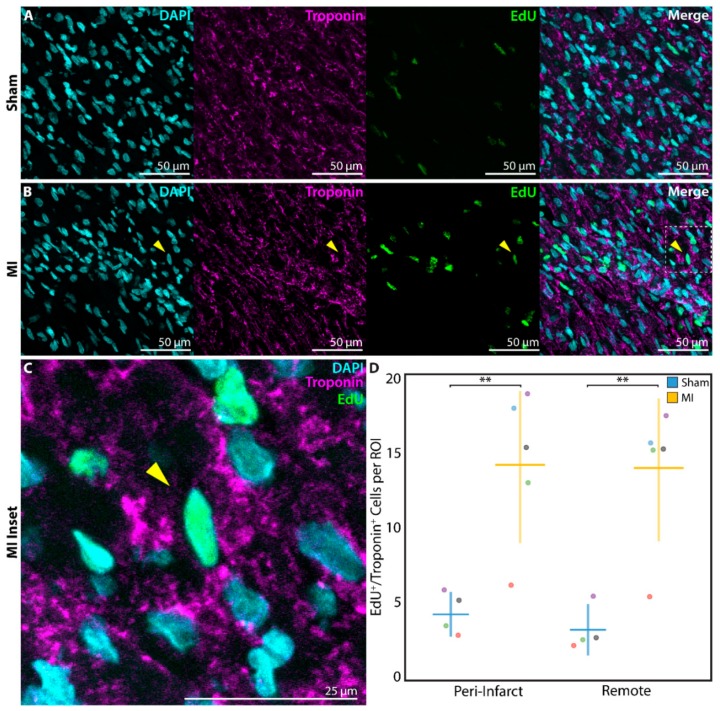
Assessment of cardiomyocyte proliferation using 5-ethynyl-2′-deoxyuridine (EdU) at 3 weeks after sham surgery (*n* = 4) or induction of myocardial infarction (MI, *n* = 5) on postnatal day 1 (P1). EdU^+^/troponin^+^ cardiomyocytes were detected within the peri-infarct region after (**A**) P1 sham and (**B**) P1 MI surgery. Inset (**C**) focuses on one EdU^+^/troponin^+^ cardiomyocyte after P1 MI, marked by the yellow arrow. (**D**) Significantly more EdU^+^/troponin^+^ cardiomyocytes were detected within each region of interest (ROI) for the P1 MI group compared to the P1 sham group in both the peri-infarct and remote regions. ** indicates *p* < 0.01. Horizontal lines indicate mean. Vertical lines indicate standard deviation.

After P1 surgery, a subset of neonatal rats in the sham (*n* = 4) and MI groups (*n* = 5) received EdU on postoperative days 1, 7, and 14, followed by heart explant at 3 weeks post-surgery. EdU, a thymidine analog and marker of DNA replication during cell proliferation [34], was found to be incorporated into the DAPI-stained nuclei of troponin^+^ cardiomyocytes at a significantly greater density in P1 MI hearts (Figure 5B,C) compared to P1 sham hearts (Figure 5A) in both the peri-infarct region (4.6 ± 1.3 cells per ROI vs. 14.1 ± 4.9 cells per ROI, *p* = 0.0072) and remote uninjured region of the LV (2.8 ± 1.5 cells per ROI vs. 13.9 ± 4.4 cells per ROI, *p* = 0.0021, Figure 5D).

**Figure 6:**
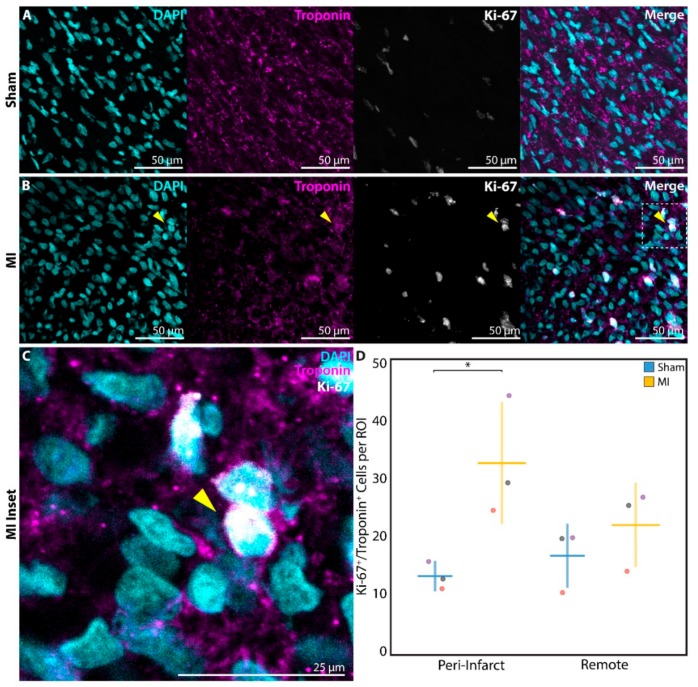
Assessment of cardiomyocyte proliferation using Ki-67 at 1 week after sham surgery (*n* = 3) or induction of myocardial infarction (MI, *n* = 3) on postnatal day 1 (P1). Ki-67^+^/troponin^+^ cardiomyocytes were detected within the peri-infarct region after (**A**) P1 sham and (**B**) P1 MI surgery. Inset (**C**) focuses on one Ki-67^+^/troponin^+^ cardiomyocyte after P1 MI, marked by the yellow arrow. (**D**) Significantly more Ki-67^+^/troponin^+^ cardiomyocytes were detected within each region of interest (ROI) for the P1 MI group compared to the P1 sham group in the peri-infarct region. * indicates *p* < 0.05. Horizontal lines indicate mean. Vertical lines indicate standard deviation.

In order to more directly assess cell cycle activation, a subset of neonatal rats in the P1 sham (*n* = 3) and P1 MI groups (*n* = 3) were sacrificed at 1 week after surgery for analysis of Ki-67 staining. Ki-67, a mitotic marker expressed in proliferating cells [35], was identified in troponin^+^ cardiomyocytes at a significantly higher frequency in P1 MI hearts (Figure 6B,C) compared to P1 sham hearts (Figure 6A) in the peri-infarct region (13.2 ± 2.3 cells per ROI vs. 32.5 ± 10.3 cells per ROI, *p* = 0.0340, Figure 6D).

**Figure 7:**
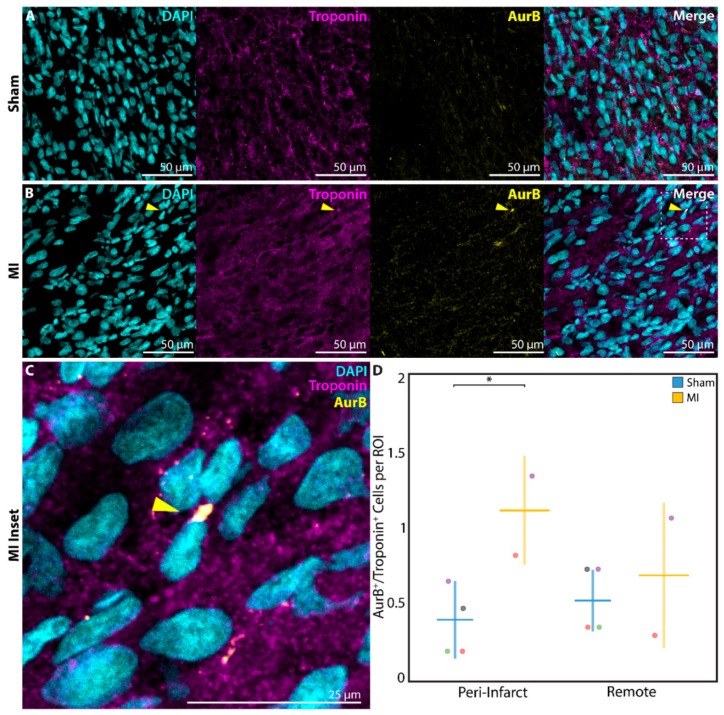
Assessment of cardiomyocyte proliferation using aurora B kinase (AurB) at 1 week after sham surgery (*n* = 4) or induction of myocardial infarction (MI, *n* = 2) on postnatal day 1 (P1). AurB^+^/troponin^+^ cardiomyocytes were detected within the peri-infarct region after (**A**) P1 sham and (**B**) P1 MI surgery. Inset (**C**) focuses on one AurB^+^/troponin^+^ cardiomyocyte after P1 MI, marked by the yellow arrow. (**D**) Significantly more AurB^+^/troponin^+^ cardiomyocytes were detected within each region of interest (ROI) for the P1 MI group compared to the P1 sham group in the peri-infarct region. * indicates *p* < 0.05. Horizontal lines indicate mean. Vertical lines indicate standard deviation.

Finally, to confirm that DNA replication and cell cycle activation resulted in cardiomyocyte proliferation, a subset of neonatal rats in the P1 sham (*n* = 4) and P1 MI (*n* = 2) groups were sacrificed at 1 week after surgery for analysis of AurB staining. AurB, which localizes to the mitotic spindle in anaphase and the cellular midbody during cytokinesis [36], was observed in significantly more troponin^+^ cardiomyocytes undergoing late mitosis or cytokinesis in P1 MI hearts (Figure 7B,C) compared to P1 sham hearts (Figure 7A) in the peri-infarct region (0.4 ± 0.3 cells per ROI vs. 1.1 ± 0.4 cells per ROI, *p* = 0.0430, Figure 7D).

### 3.5. Cardiac Geometry and Function Fully Recover by 3 Weeks after Myocardial Infarction

Of the 74 rats in our study, 56 were intended for 3-week post-surgical follow up. Overall, 47/56 rats survived (83.9%) to the 3-week timepoint, including 95.7% survival (22/23) among rats in the sham group and 75.8% survival (25/33) among rats in the MI group. The majority of losses occurred during the immediate days after P1 surgery. For adult rats in the sham and MI groups (*n* = 3, each), 3-week postoperative survival was 100%.

At 3 weeks after sham or LAD ligation surgery, neonate and adult rats underwent echocardiography to assess cardiac size and function (Table 2). Among the P1 sham (*n* = 22) and P1 MI rats (*n* = 25), no significant differences were observed in LVWTd (0.91 mm vs. 0.91 mm, *p* = 0.9458), LVIDs (2.68 mm vs. 2.76 mm, *p* = 0.3592), LVIDd (4.50 mm vs. 4.52 mm, *p* = 0.7801), EF (71.1% vs. 69.2%, *p* = 0.2511), stroke volume (66.2 μL vs. 65.0 μL, *p* = 0.7259), or cardiac output (26.3 mL/min vs. 26.7 mL/min, *p* = 0.7652), respectively. When the echocardiography data was analyzed for female neonatal rats (sham *n* = 6, MI *n* = 13) and male neonatal rats (sham *n* = 16, MI *n* = 12) separately, again no differences were observed for any parameter. Compared to adult sham rats (*n* = 3), adult MI rats (*n* = 3) had hearts which were significantly thinned (LVWTd 1.52 mm vs. 0.82 mm, *p* = 0.0008) and dilated (LVIDs 4.93 mm vs. 8.46 mm, *p* < 0.0001; LVIDd 8.74 mm vs. 10.26 mm, *p* = 0.0078), with dramatically reduced cardiac function (EF 72.5% vs. 34.4%, *p* = 0.0001; cardiac output 117.1 mL/min vs. 73.6 mL/min, *p* = 0.0078).

**Figure 8:**
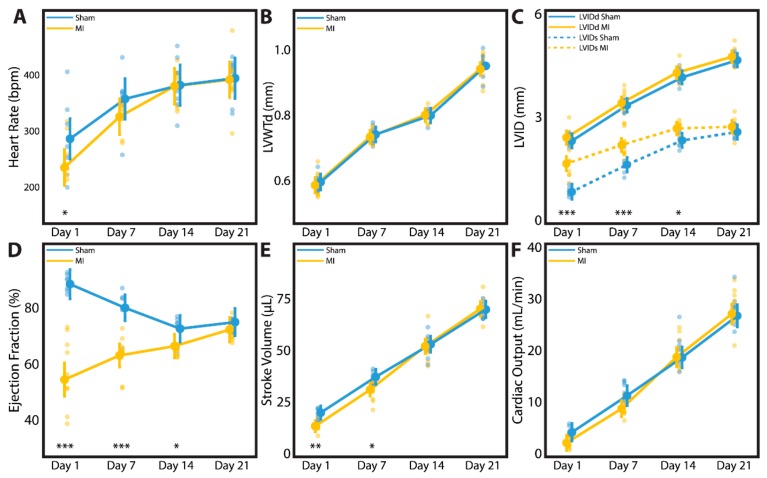
Time-course echocardiography was performed for neonatal rats at 1 day, 1 week, 2 weeks, and 3 weeks after sham surgery (*n* = 7) or induction of myocardial infarction (MI, *n* = 9) on postnatal day 1. Parameters studied include (**A**) heart rate, (**B**) left ventricular wall thickness in diastole (LVWTd), (**C**) left ventricular internal diameter in diastole (LVIDd) and systole (LVIDs), (**D**) ejection fraction, (**E**) stroke volume, and (**F**) cardiac output. * indicates *p* < 0.05. ** indicates *p* < 0.01. *** indicates *p* < 0.001. Large dots indicate mean. Small dots represent individual data points. Vertical lines indicate 95% confidence interval.

A subset of neonatal rats followed to 3 weeks post-surgery underwent time-course echocardiographic studies at 1 day, 1 week, 2 weeks, and 3 weeks after sham surgery (*n* = 7) or LAD ligation (*n* = 9). Data regarding heart rate, LVWTd, LVIDs, LVIDd, EF, stroke volume, and cardiac output are summarized for each individual animal and for the P1 sham and P1 MI subcohorts in Table S1. Because each of these animals underwent echocardiography at multiple timepoints, a mixed-model analysis was performed to account for clustering effects within each individual animal and to adjust for heart rate as a potential confounding variable. Indeed, the P1 MI subcohort had a slower heart rate compared to the P1 sham subcohort at 1 day after surgery (285.1 bpm vs. 233.0 bpm, *p* = 0.0383). Within 1–2 weeks after surgery, however, the heart rate of the P1 MI subcohort had normalized to equal that of the P1 sham subcohort (Figure 8A). Therefore, it was important to include heart rate as a covariate in our mixed-model analysis to correct for any confounding effect on other echocardiographic metrics.

At 3 weeks post-surgery, there was no difference between the sham and MI subcohorts in terms of body weight (61.4 ± 4.1 g vs. 59.7 ± 4.0 g, *p* = 0.4026), explanted heart weight (0.32 ± 0.02 g vs. 0.32 ± 0.02 g, *p* = 0.6346), or heart-body weight ratio (0.53 ± 0.03% vs. 0.53 ± 0.05%, *p* = 0.7075). In terms of cardiac geometry, LVWTd (Figure 8B) and LVIDd (Figure 8C) were similar between the sham and MI subcohorts throughout all timepoints assessed, confirming the absence of adverse LV remodeling after P1 MI. Nevertheless, all rats in the P1 MI subcohort had confirmed LV hypokinesis at 1 day post-MI (LVIDs sham 0.98 mm vs. MI 1.74 mm, *p* < 0.0001), and this difference persisted at 1 week (1.72 mm vs. 2.26 mm, *p* = 0.0003) and 2 weeks after LAD ligation (2.38 mm vs. 2.71 mm, *p* = 0.0205, Figure 8C). By 3 weeks post-MI however, LVIDs was similar between the sham and MI subcohorts (2.62 mm vs. 2.75 mm, *p* = 0.3001). In concordance, the mean EF of the P1 MI subcohort at 1 day after surgery was reduced to 57.2%. By 3 weeks after surgery, however, LV function improved significantly within the P1 MI subcohort to a mean EF of 71.7% (*p* < 0.0001). In contrast, hyperdynamic LV function was observed in the P1 sham subcohort at 1 day after surgery (mean EF 89.6%), and a gradual decrease in mean EF to 73.8% was noted by 3 weeks after surgery (*p* = 0.0012). While the P1 sham and P1 MI subcohorts exhibited significantly different EF at 1 day (89.6% vs. 57.2%, *p* < 0.0001), 1 week (80.3% vs. 63.3%, *p* < 0.0001), and 2 weeks after surgery (72.4% vs. 65.6%, *p* = 0.0478), the two groups had comparable EF at 3 weeks after surgery (73.8% vs. 71.7%, *p* = 0.3858, Figure 8D). This gradual recovery in LV function after P1 MI was correlated with normalization of stroke volume and cardiac output. While the P1 MI subcohort had reduced stroke volume and cardiac output compared to the P1 sham subcohort at 1 day (17.6 μL vs. 11.8 μL, *p* = 0.0085; 4.9 mL/min vs. 2.8 mL/min, *p* = 0.0944) and 1 week after surgery (36.3 μL vs. 29.7 μL, *p* = 0.0166; 12.9 mL/min vs. 9.6 mL/min, *p* = 0.0533), the two groups had similar stroke volume and cardiac output at 2 weeks (52.7 μL vs. 52.1 μL, *p* = 0.7039; 20.1 mL/min vs. 19.8 mL/min, *p* = 0.9562) and 3 weeks after surgery (70.7 μL vs. 71.6 μL, *p* = 0.9210; 27.9 mL/min vs. 28.0 mL/min, *p* = 0.8163, Figure 8E,F).

## 4. Discussion

Our study introduces a neonatal rat MI model involving permanent LAD ligation performed while the animal is under hypothermic circulatory arrest. Using this model, we demonstrate that neonatal rats at age P1 are capable of natural heart regeneration after MI, resulting in minimal LV scar formation, as well as preservation of normal cardiac size and LV function despite ischemic injury. These results build upon previous observations that suggest that natural heart regeneration may be a conserved phenotype among many newborn mammals, possibly including humans [37].

Neonatal heart regeneration was first demonstrated in 2011 using an apical resection model in P1 mice [22]. Similar observations have also been reported in P1 rats after apical resection [23]. This injury model is focused on cardiomyocyte replacement in a mechanically disrupted cardiac apex, which provides compelling evidence for new tissue growth, but is not clinically or translationally relevant. The MI injury model, however, replicates a disease process that remains one of the leading causes of death worldwide for mankind. Developing a regeneration-based solution for post-MI ischemic cardiomyopathy has the potential to save millions of lives each year [1].

Among mammalian research models, natural neonatal heart regeneration after MI has previously only been confirmed in mice and piglets [24,25,26,27,28]. Although substantial insights regarding natural heart regeneration have been gleaned from the neonatal murine model, several notable limitations of this small animal model may impede widespread adoption and experimental reproducibility. Due to the very small size of the P1 mouse heart (approximately 1–2 mm diameter), the learning curve for developing expertise and consistency with microsurgery for the neonatal mouse MI model can be formidable [37]. Tearing of the fragile LV tissue and the risk of fatal bleeding given the extremely low blood volume of the P1 mouse (<100 μL) [38] are among the technical challenges related to the small size of the P1 mouse heart. Moreover, maternal cannibalization is a major concern after neonatal mouse surgery. Indeed, the expected immediate postoperative survival rate for P1 mouse surgery ranges from 70%–90%, but this figure is commonly reduced to 60%–70% by 3 weeks after surgery due to maternal cannibalization [31].

To perform LAD ligation in neonatal rats, we applied our experience with topical cooling and hypothermic circulatory arrest in neonatal mice [26,39] to an upscaled rat model. We observed that P1 rats required 7 min of cooling on ice to induce apnea and asystole that reproducibly lasted the duration of surgery. Under this hypothermic anesthesia, the neonatal rats tolerated an open thoracotomy without mechanical ventilation and suffered minimal bleeding due to the heart being arrested during the procedure. The LV tissue of neonatal rats was structurally stronger than that of neonatal mice, tolerating needle passes without tearing. The larger size of the P1 rat heart (approximately 3–4 mm diameter) simplified the technical difficulty of the surgical procedure considerably. As a result, we were able to perform LAD ligation in neonatal rats in a high-throughput manner (5–6 min per surgery), and with >97% operative survival. Importantly, we observed only one case of maternal cannibalization postoperatively, in which the mother immediately began to kill the pups that were returned to her nest after surgery. Transferring the survivors to a surrogate mother with age-matched pups proved successful in this case. At the 3-week study endpoint, we recorded a survival rate of 83.9%. Overall, these data support the viability of using neonatal rats as an alternative small animal model for studying natural heart regeneration after MI.

Previous genetic lineage tracing experiments have demonstrated that, in neonatal mouse models of natural heart regeneration, new cardiomyocytes in regenerated cardiac tissue were derived from preexisting cardiomyocytes [22,24,25]. To investigate cardiomyocyte cell proliferation in this study, we performed a pulse-chase experiment with EdU given at 1, 7, and 14 days post-MI. These timepoints were determined on the basis of cardiomyocyte binucleation occurring between ages P4–P12 in rats [40], thus ensuring that we captured EdU^+^ proliferating cardiomyocytes prior to binucleation. Consistent with our time-course echocardiographic analyses, which revealed that natural heart regeneration after LAD ligation in P1 rats occurs during the first few weeks after ischemic injury, we observed a significant increase in EdU^+^/troponin^+^ cardiomyocytes within both the peri-infarct and remote regions of the LV during this timeframe. Furthermore, we also observed significantly more Ki-67^+^/troponin^+^ and AurB^+^/troponin^+^ cardiomyocytes within the peri-infarct region of the LV at 1 week post-MI as well. These data collectively suggest an increase not only in DNA replication and cell cycle activation but also in cytokinesis and cardiomyocyte proliferation during the first 1–2 weeks after P1 MI. This process results in gradual myocardial regeneration and complete LV functional recovery by 3 weeks post-MI. Future studies aimed at elucidating the mechanisms involved with natural heart regeneration are necessary, including the role of angiogenesis [26,39,41], the immune system [42,43], the endocrine system [44], and the nervous system [45].

Our study is subject to some important limitations. As previously noted, inconsistent infarct size is a major drawback of any LAD ligation model. This is especially true in the neonatal MI model where LAD ligation is performed on an arrested heart, preventing direct visualization of LV pallor and hypokinesis. In our study, we assessed cardiac function at 1 day after LAD ligation surgery in 22 neonatal rats, and only 2 (9.1%) did not exhibit any evidence of anterolateral wall hypokinesis. Thus, we report a procedural success rate of 90.9%. However, of the remaining 20 rats that exhibited definite anterolateral wall hypokinesis, the range in measured EF was 38.3% to 73.4%, with a mean of 53.0%. It is important to note that, relative to the hyperdynamic function of the normal neonatal rat heart at this age (mean EF 87.1%), an EF of 73% still represents a state of LV dysfunction. Nevertheless, several factors may have contributed to the variability observed in EF at 1 day after P1 MI. It is possible that cardiac function was reduced in some animals during imaging due to excessive inhaled isoflurane anesthetic [46]. Indeed, the heart rate of the MI group at 1 day after P1 surgery was significantly slower than that of the sham group. However, we demonstrated no direct correlation between heart rate and EF in either group at this age, and we also confirmed using an advanced statistical model that the observed differences in EF and other echocardiographic metrics between the sham and MI groups at 1 day through 3 weeks after P1 surgery were independent of heart rate. These results suggest that anesthesia-induced cardiac depression was not the cause of low EF in the P1 MI group. Alternatively, it is possible that some hearts received a higher-level ligation on the LAD than others. Large-scale injury to the myocardium has been reported to result in incomplete regeneration [47,48]. In our study, however, of the aforementioned 20 rats that exhibited anterolateral wall hypokinesis at 1 day after P1 MI (EF range 38.3% to 73.4%, mean 53.0%), at 3 weeks post-MI the range of EF was 67.6% to 78.6% (mean 71.7%). This degree of heart function was similar to that of sham hearts and the narrow range reflects a consistent recovery to a normal contractile state. Furthermore, the P1 MI hearts selected for collagen scar analysis had 1-day postoperative EFs of 38.3%, 51.3%, 56.2%, and 73.4%, but all of these hearts exhibited approximately the same degree of fibrotic scar at 3 weeks post-MI (range 1.15% to 1.75% of LV area on Masson’s trichome analysis, all of which were greater than the mean 0.68% of LV area observed in the sham group). These results altogether indicate that, although perfect histological recovery was not observed at 3 weeks after P1 MI in neonatal rats, complete functional recovery and preservation of LV geometry was observed in all cases. 

Our study also features several important strengths. Unlike many previous studies of natural heart regeneration after MI, we ensured that our sham control group received the mechanical damage associated with needle insertion during LAD ligation. As a result, we can conclude that our observations regarding myocardial regeneration are secondary to ischemic injury. This is important because mechanical injury, as evident from heart regeneration studies using apical resection, can also induce cardiomyocyte proliferation. Furthermore, we also examined sex-specific functional outcomes of post-MI cardiac regeneration, which revealed that both males and females were capable of natural heart regeneration after P1 MI. Finally, we performed time-course echocardiography in a subset of rats after P1 surgery, which not only ensured that all rats in the MI subcohort had definite LV hypokinesis after LAD ligation, but also that a clear recovery in LV function was observed for each P1 MI rat over the first 3 weeks after surgery.

## 5. Conclusions

We describe a neonatal rat MI model that may serve as a valuable resource for the study of natural heart regeneration. P1 rats are capable of endogenous neocardiomyogenesis after MI, adding further evidence in support of the theory that natural heart regeneration may be a conserved phenotype among newborn mammals.

## Figures and Tables

**Table 1:** Echocardiography assessment at Day 1 after surgery.

P1 Rats: Day 1 After Surgery	P1 Sham (*n* = 15)	P1 MI (*n* = 20)	*p*-Value
Heart Rate (bpm)	290.2 ± 57.0	238.2 ± 43.3	0.0042
LVWTd (mm)	0.59 ± 0.02	0.58 ± 0.03	0.5343
LVIDs (mm)	1.10 ± 0.21	1.86 ± 0.33	<0.0001
LVIDd (mm)	2.44 ± 0.21	2.51 ± 0.27	0.4551
Ejection Fraction (%)	87.1 ± 4.5	53.0 ± 10.4	<0.0001
Stroke Volume (μL)	18.5 ± 3.6	11.8 ± 2.4	<0.0001
Cardiac Output (mL/min)	5.3 ± 1.2	2.8 ± 0.8	<0.0001

Mean and standard deviation are summarized for each group. LVIDd, left ventricle internal diameter in diastole; LVIDs, left ventricle internal diameter in systole; LVWTd, left ventricle wall thickness in diastole; MI, myocardial infarction; P1, postnatal day 1.

**Table 2:** Echocardiography assessment at Week 3 after surgery.

P1 Rats: Week 3 After Surgery	P1 Sham (*n* = 22)	P1 MI (*n* = 25)	*p*-Value
Heart Rate (bpm)	399.4 ± 51.8	408.7 ± 43.8	0.5060
Female	399.7 ± 43.7	394.8 ± 41.9	0.8181
Male	399.3 ± 55.9	423.8 ± 42.3	0.2147
LVWTd (mm)	0.91 ± 0.05	0.91 ± 0.06	0.9458
Female	0.92 ± 0.05	0.92 ± 0.06	0.9327
Male	0.90 ± 0.06	0.90 ± 0.05	0.8508
LVIDs (mm)	2.68 ± 0.26	2.76 ± 0.29	0.3592
Female	2.66 ± 0.29	2.76 ± 0.30	0.5114
Male	2.69 ± 0.26	2.75 ± 0.29	0.5462
LVIDd (mm)	4.50 ± 0.29	4.52 ± 0.26	0.7801
Female	4.43 ± 0.30	4.52 ± 0.32	0.5546
Male	4.53 ± 0.29	4.52 ± 0.20	0.9619
Ejection Fraction (%)	71.1 ± 4.3	69.2 ± 6.3	0.2511
Female	70.7 ± 4.2	69.1 ± 7.4	0.6275
Male	71.3 ± 4.5	69.4 ± 5.3	0.3271
Stroke Volume (μL)	66.2 ± 13.7	65.0 ± 10.5	0.7259
Female	62.7 ± 13.1	66.4 ± 12.8	0.5609
Male	67.5 ± 14.1	63.4 ± 7.5	0.3622
Cardiac Output (mL/min)	26.3 ± 4.6	26.7 ± 4.0	0.7652
Female	25.1 ± 3.0	26.0 ± 4.7	0.6750
Male	26.8 ± 5.1	27.5 ± 3.2	0.6743
**Adult Rats:** **Week 3 After Surgery**	**Adult Sham** **(*n* = 3)**	**Adult MI** **(*n* = 3)**	***p*-value**
Heart Rate (bpm)	384.5 ± 15.8	360 ± 41.9	0.3969
LVWTd (mm)	1.52 ± 0.11	0.82 ± 0.07	0.0008
LVIDs (mm)	4.93 ± 0.09	8.46 ± 0.18	<0.0001
LVIDd (mm)	8.74 ± 0.30	10.26 ± 0.44	0.0078
Ejection Fraction (%)	72.5 ± 2.2	34.4 ± 3.2	0.0001
Stroke Volume (μL)	290.0 ± 58.9	197.1 ± 54.5	0.1158
Cardiac Output (mL/min)	117.1 ± 13.3	73.6 ± 7.3	0.0078

Mean and standard deviation are summarized for each group. Among P1 sham rats, females and males accounted for 6 and 16 animals, respectively. Among P1 MI rats, females and males accounted for 13 and 12 animals, respectively. All adult rats used in this study were male. LVIDd, left ventricle internal diameter in diastole; LVIDs, left ventricle internal diameter in systole; LVWTd, left ventricle wall thickness in diastole; MI, myocardial infarction; P1, postnatal day 1.

## Supplementary Materials

The following are available online at https://www.mdpi.com/2073-4409/9/1/229/s1: Figure S1, Relationship of ejection fraction versus heart rate; Table S1, Time-course echocardiography assessment after P1 surgery; Video S1, Neonatal rat myocardial infarction model.
