# Supplementary material for: Natural Heart Regeneration in a Neonatal Rat Myocardial Infarction Model
Source: Cells. 2020 Jan 16;9(1):229. doi: 10.3390/cells9010229 (PMC7017245; doi:10.3390/cells9010229)
Supplement: Supplementary file 1 [file cells-09-00229-s001.zip › Supplemental Figure and Table FINAL.docx]

**Figure S1.** Relationship of ejection fraction versus heart rate. Neonatal rats in the sham (blue) and myocardial infarction (MI, yellow) groups underwent echocardiography at 1 day after surgery performed on postnatal day 1. No direct correlation is observed between heart rate and ejection fraction.

**Table S1.** Time-course echocardiography assessment after P1 surgery.

| **Echocardiography** | **P1 Sham (n=7)** | 1 | 2 | 3 | 4 | 5 | 6 | 7 | **P1 MI (n=9)** | 1 | 2 | 3 | 4 | 5 | 6 | 7 | 8 | 9 | **P-value** |
| --- | --- | --- | --- | --- | --- | --- | --- | --- | --- | --- | --- | --- | --- | --- | --- | --- | --- | --- | --- |
| **HR (bpm)** | | | | | | | | | | | | | | | | | | | |
| **Day 1** | **285.1 ± 72.7** | 220.0 | 271.0 | 197.0 | 251.0 | 338.0 | 312.0 | 407.0 | **233.0 ± 55.2** | 154.0 | 164.0 | 256.0 | 240.0 | 220.0 | 211.0 | 231.0 | 317.0 | 304.0 | **0.0383** |
| **Week 1** | **357.7 ± 52.6** | 346.0 | 367.0 | 433.0 | 370.0 | 372.0 | 360.0 | 256.0 | **325.3 ± 27.6** | 350.0 | 335.0 | 321.0 | 326.0 | 340.0 | 278.0 | 351.0 | 345.0 | 282.0 | **0.1931** |
| **Week 2** | **382.7 ± 50.8** | 309.0 | 433.0 | 454.0 | 358.0 | 405.0 | 348.0 | 372.0 | **380.9 ± 33.4** | 378.0 | 400.0 | 404.0 | 393.0 | 428.0 | 340.0 | 405.0 | 335.0 | 345.0 | **0.9411** |
| **Week 3** | **395.3 ± 42.4** | 407.0 | 423.0 | 331.0 | 415.0 | 338.0 | 434.0 | 419.0 | **392.6 ± 49.1** | 380.0 | 412.0 | 407.0 | 397.0 | 482.0 | 373.0 | 411.0 | 295.0 | 376.0 | **0.9120** |
| **LVWTd (mm)** | | | | | | | | | | | | | | | | | | | |
| **Day 1** | **0.59 ± 0.03** | 0.60 | 0.56 | 0.57 | 0.60 | 0.64 | 0.56 | 0.58 | **0.58 ± 0.04** | 0.55 | 0.61 | 0.62 | 0.55 | 0.57 | 0.54 | 0.56 | 0.65 | 0.55 | **0.5811** |
| **Week 1** | **0.74 ± 0.03** | 0.70 | 0.75 | 0.71 | 0.77 | 0.78 | 0.72 | 0.75 | **0.73 ± 0.02** | 0.74 | 0.72 | 0.75 | 0.71 | 0.71 | 0.70 | 0.73 | 0.76 | 0.77 | **0.6463** |
| **Week 2** | **0.80 ± 0.02** | 0.76 | 0.80 | 0.79 | 0.80 | 0.81 | 0.80 | 0.82 | **0.80 ± 0.02** | 0.79 | 0.80 | 0.82 | 0.81 | 0.77 | 0.77 | 0.80 | 0.84 | 0.82 | **0.9364** |
| **Week 3** | **0.96 ± 0.04** | 0.96 | 0.97 | 0.89 | 0.95 | 0.99 | 0.92 | 1.01 | **0.95 ± 0.04** | 0.98 | 0.97 | 0.93 | 0.90 | 0.94 | 0.99 | 0.88 | 1.00 | 0.95 | **0.5321** |
| **LVIDs (mm)** | | | | | | | | | | | | | | | | | | | |
| **Day 1** | **0.98 ± 0.12** | 0.91 | 0.83 | 1.17 | 0.91 | 0.97 | 1.00 | 1.07 | **1.74 ± 0.38** | 1.44 | 1.80 | 1.44 | 1.54 | 1.84 | 2.11 | 1.22 | 1.80 | 2.46 | **<0.0001** |
| **Week 1** | **1.72 ± 0.27** | 1.51 | 2.21 | 1.36 | 1.73 | 1.74 | 1.83 | 1.66 | **2.26 ± 0.36** | 2.04 | 2.09 | 2.37 | 2.86 | 2.77 | 2.33 | 1.83 | 2.00 | 2.02 | **0.0003** |
| **Week 2** | **2.38 ± 0.20** | 2.39 | 2.10 | 2.24 | 2.25 | 2.67 | 2.44 | 2.60 | **2.71 ± 0.20** | 2.53 | 2.84 | 2.33 | 2.79 | 2.84 | 2.79 | 2.53 | 2.78 | 2.93 | **0.0205** |
| **Week 3** | **2.62 ± 0.17** | 2.50 | 2.44 | 2.79 | 2.62 | 2.72 | 2.84 | 2.42 | **2.75 ± 0.25** | 2.30 | 2.81 | 2.64 | 3.17 | 2.56 | 2.85 | 2.83 | 2.63 | 2.95 | **0.3001** |
| **LVIDd (mm)** | | | | | | | | | | | | | | | | | | | |
| **Day 1** | **2.36 ± 0.15** | 2.46 | 2.35 | 2.56 | 2.28 | 2.15 | 2.51 | 2.24 | **2.43 ± 0.32** | 2.42 | 2.39 | 2.21 | 2.56 | 2.44 | 2.60 | 1.80 | 2.49 | 2.99 | **0.5244** |
| **Week 1** | **3.33 ± 0.15** | 3.13 | 3.45 | 3.10 | 3.34 | 3.38 | 3.47 | 3.44 | **3.39 ± 0.34** | 3.09 | 3.15 | 3.64 | 3.87 | 3.73 | 3.56 | 2.81 | 3.38 | 3.24 | **0.6258** |
| **Week 2** | **4.10 ± 0.23** | 4.42 | 3.77 | 4.11 | 3.95 | 4.19 | 4.31 | 3.94 | **4.21 ± 0.25** | 3.89 | 4.27 | 3.98 | 4.34 | 4.39 | 4.20 | 3.94 | 4.22 | 4.68 | **0.3356** |
| **Week 3** | **4.57 ± 0.16** | 4.54 | 4.41 | 4.73 | 4.50 | 4.59 | 4.81 | 4.40 | **4.65 ± 0.24** | 4.35 | 4.66 | 4.50 | 5.09 | 4.37 | 4.65 | 4.78 | 4.57 | 4.87 | **0.4659** |
| **EF (%)** | | | | | | | | | | | | | | | | | | | |
| **Day 1** | **89.6 ± 3.1** | 92.5 | 93.3 | 86.7 | 90.9 | 87.4 | 90.8 | 85.3 | **57.2 ± 12.9** | 73.4 | 51.3 | 67.0 | 72.5 | 51.0 | 40.8 | 64.2 | 56.2 | 38.3 | **<0.0001** |
| **Week 1** | **80.3 ± 7.1** | 84.2 | 65.6 | 87.6 | 80.5 | 80.7 | 79.7 | 83.8 | **63.3 ± 7.3** | 64.4 | 63.9 | 65.1 | 51.7 | 51.3 | 64.5 | 66.2 | 73.0 | 69.3 | **<0.0001** |
| **Week 2** | **72.4 ± 5.2** | 73.7 | 76.2 | 77.3 | 74.8 | 66.3 | 74.9 | 63.8 | **65.6 ± 3.2** | 64.8 | 62.5 | 72.8 | 65.6 | 65.0 | 62.6 | 66.0 | 63.4 | 67.4 | **0.0478** |
| **Week 3** | **73.8 ± 2.3** | 76.1 | 76.2 | 72.0 | 72.8 | 71.6 | 71.7 | 76.5 | **71.7 ± 3.3** | 78.6 | 70.1 | 72.5 | 67.6 | 72.6 | 68.9 | 71.3 | 73.8 | 69.6 | **0.3858** |
| **SV (μL)** | | | | | | | | | | | | | | | | | | | |
| **Day 1** | **17.6 ± 2.9** | 19.9 | 17.9 | 20.7 | 16.2 | 13.4 | 20.4 | 14.5 | **11.8 ± 3.1** | 15.1 | 10.3 | 11.1 | 17.1 | 10.8 | 10.1 | 6.2 | 12.4 | 13.3 | **0.0085** |
| **Week 1** | **36.3 ± 3.4** | 32.7 | 32.9 | 33.3 | 36.6 | 37.6 | 39.7 | 41.0 | **29.7 ± 5.6** | 24.3 | 25.3 | 36.4 | 33.5 | 30.5 | 34.3 | 19.8 | 34.2 | 29.3 | **0.0166** |
| **Week 2** | **52.7 ± 6.8** | 56.2 | 46.4 | 57.8 | 50.6 | 51.8 | 62.6 | 43.1 | **52.1 ± 7.6** | 42.5 | 51.0 | 50.5 | 55.8 | 56.8 | 49.3 | 44.6 | 50.4 | 68.3 | **0.7039** |
| **Week 3** | **70.7 ± 4.2** | 71.8 | 67.1 | 75.0 | 67.2 | 69.5 | 77.6 | 67.0 | **71.6 ± 6.3** | 67.3 | 70.3 | 67.2 | 83.6 | 62.8 | 68.8 | 75.9 | 70.8 | 77.5 | **0.9210** |
| **CO (mL/min)** | | | | | | | | | | | | | | | | | | | |
| **Day 1** | **4.9 ± 0.9** | 4.4 | 4.8 | 4.1 | 4.1 | 4.5 | 6.4 | 5.9 | **2.8 ± 1.0** | 2.3 | 1.7 | 2.8 | 4.1 | 2.4 | 2.1 | 1.4 | 3.9 | 4.1 | **0.0944** |
| **Week 1** | **12.9 ± 1.6** | 11.3 | 12.1 | 14.4 | 13.6 | 14.0 | 14.3 | 10.5 | **9.6 ± 1.7** | 8.5 | 8.5 | 11.7 | 10.9 | 10.4 | 9.5 | 6.9 | 11.8 | 8.2 | **0.0533** |
| **Week 2** | **20.1 ± 3.4** | 17.4 | 20.1 | 26.2 | 18.1 | 21.0 | 21.8 | 16.0 | **19.8 ± 3.1** | 16.0 | 20.4 | 20.4 | 21.9 | 24.3 | 16.8 | 18.1 | 16.9 | 23.6 | **0.9562** |
| **Week 3** | **27.9 ± 3.3** | 29.2 | 28.4 | 24.8 | 27.9 | 23.5 | 33.7 | 28.1 | **28.0 ± 3.6** | 25.5 | 29.0 | 27.4 | 33.1 | 30.3 | 25.6 | 31.2 | 20.9 | 29.2 | **0.8163** |

Mean and standard deviation are summarized in bold for each group at each timepoint. Individual data are presented at each timepoint for all animals. P-values reflect post-hoc analyses after mixed-model adjustments. CO, cardiac output; EF, ejection fraction; LVIDd, left ventricle internal diameter in diastole; LVIDs, left ventricle internal diameter in systole; LVWTd, left ventricle wall thickness in diastole; MI, myocardial infarction; P1, postnatal day 1; SV, stroke volume.
